# Fbw7/hCDC4 dimerization regulates its substrate interactions

**DOI:** 10.1186/1747-1028-2-7

**Published:** 2007-02-13

**Authors:** Markus Welcker, Bruce E Clurman

**Affiliations:** 1 Division of Human Biology, Fred Hutchinson Cancer Research Center, 1100 Fairview Ave N, Seattle, Washington, 98109, USA; 2 Clinical Research Division, Fred Hutchinson Cancer Research Center, 1100 Fairview Ave N, Seattle, Washington, 98109, USA; 3 Department of Medicine, University of Washington School of Medicine, Seattle, Washington, 98104, USA

## Abstract

**Background:**

The Fbw7 ubiquitin ligase promotes the rapid degradation of several important oncogenes, such as cyclin E, c-Myc, c-Jun, and Notch. The two fission yeast homologs of Fbw7, pop1 and pop2, have previously been shown to dimerize. In this study, we asked whether Fbw7 can also dimerize and how dimerization affects Fbw7 function.

**Results:**

We found that Fbw7 binds efficiently to itself through a domain just upstream of its F-box. We further show that dimerization is essential for the stable interaction of Fbw7 with the cyclin E T380 phospho-degron. Surprisingly, the requirement for dimerization can be suppressed by an additional phosphorylation of this phospho-degron at the +4 position (S384), which creates a binding site with higher affinity for monomeric Fbw7.

**Conclusion:**

Degradation of cyclin E by the Fbw7 pathway can, thus, be conditionally regulated either by Fbw7 dimerization or by hyperphosphorylation of the T380 phospho-degron. Other substrates, which cannot accommodate an extra phosphate in their phospho-degrons, or which don't provide a negatively charged amino acid in the +4 position, may be absolutely dependent on Fbw7 dimerization for their turnover. Our results point to an additional level of regulation for substrate interaction and turnover by Fbw7.

## Background

Fbw7 is the mammalian homolog of budding yeast CDC4 and mediates the degradation of several proteins involved in cell growth and division, including cyclin E, c-Myc, c-Jun, Notch, Presenilin, and SREBP [1-10]. Fbw7 recognizes a phospho-epitope, termed CPD (for Cdc4 Phospho-Degron), contained within these substrates. Via its F-box, Fbw7 recruits the remainder of an SCF ubiquitin ligase complex, thus promoting substrate ubiquitination and rapid degradation by the proteasome [11]. Mammalian cells contain three Fbw7 isoforms (Fbw7α, Fbw7β, and Fbw7γ) that are produced by alternative splicing and that localize to the nucleoplasm, cytoplasm, and nucleolus, respectively [12-14].

Numerous cancer-associated mutations have been identified within CPDs of Fbw7 substrates that render them insensitive to Fbw7 regulation. Accordingly, the Fbw7 gene is deleted in a large number of tumors. Moreover, many somatic point mutations have been found that eliminate Fbw7's function either by terminating the protein prematurely or disabling its substrate recognition domain, the C-terminal WD40 repeats [15].

The eight WD40 repeats form a beta-propeller structure creating a phospho-epitope binding pocket that can recognize phosphorylated CPDs [16]. All currently known mammalian CPDs consist of a central phosphorylated threonine immediately followed by proline (pT-P). For substrates like c-Myc, c-Jun, SREBP, and possibly cyclin E, a phosphorylated serine in the +4 position serves as priming phosphate for GSK3 in order to phosphorylate the central threonine. However, beyond simply priming for GSK3, there appear to be additional requirements for this negative charge in the +4 position for substrate turnover by Fbw7.

The first evidence for this role of the +4 negative charge came from studies of cyclin E degradation. Cyclin E's turnover by Fbw7 depends on the phosphorylation of T380, the central phospho-threonine [2,3,17,18]. We previously identified S384 as a second phosphorylation site that plays a key role in the formation of this CPD [19,20]. However, we noticed that degradation of cyclin E by either Fbw7β or Fbw7γ strictly required S384 phosphorylation, whereas the requirement for this phosphorylation by Fbw7α was depended on the stoichiometry of Fbw7α and cyclin E. That is, high Fbw7α abundance relative to cyclin E can overcome the need for S384 phosphorylation. Moreover, an S384 glutamate mutant (S384E) is degraded like wild-type cyclin E, emphasizing the importance of a negative charge rather than a (priming) phosphate in this position. Further evidence for a role of the +4 negative charge was provided by the interaction between Fbw7 and the SV40 Large T oncoprotein, which also binds to Fbw7 via a CPD and interferes with Fbw7 function [21]. Large T contains a glutamate in the +4 position, and this residue is crucial for its interaction with Fbw7. In fact, almost every substrate of Fbw7 and its homologs potentially provides a negative charge in the +4 position in form of either phosphoserine/threonine or a negatively charged glutamate (Table 1) [3-5,10,17,18,21-27]. Thus, the +4 negative charge is highly conserved and likely plays a central role in substrate regulation. Ironically, one exception to this rule is the best-studied CDC4 substrate, Sic1, which contains a number of imperfect CPDs that interact allovalently with CDC4 [16]. However, for CDC4 binding to CPDs, the -1 and -2 positions have also been shown to be important instead of +4, generating a slightly different consensus: I/L-I/L/P-pTP <K/R>4 where <K/R> are disfavored residues in positions +2 to +5 [11]. Although cyclin E's T380 degron completely conforms with this consensus we observed the additional requirement of S384 phosphorylation for its turnover by Fbw7.

**Table 1:** Alignment of CPDs.

***Conservation of the negative charge in the +4 position of CPDs***				
		0 +4		references
				
cyclin E:	T380	LL**T**PPQ**S**GK	mammalian	(1–3)
	T62	IP**T**PDK**E**DD		(3)
c-Myc :	T58	LP**T**PPL**S**PS		(4,5)
c-Jun:	T239	GE**T**PPL**S**PI		(22)
SREBP 1:	T456*	TL**T**PPP**S**DA		(10)
SV40/Large T:	T701	PP**T**PPP**E**PE		(21)
Notch 1:	T2512*	FL**T**PSP**E**SP		
Presenilin 1:	T116	IY**T**PFT**E**DT		
Gcn4:	T165	LP**T**PVL**E**DA	yeast	(23)
Far1 :	S87	PI**S**PPP**S**LK		(24)
Cdc6:	T39	DV**T**PES**S**PE		(25)
	T368	PL**T**PTT**S**PV		(25)
Clb6:	S6	IP**S**PIS**E**RK		(26)
	T39	NL**T**PHS**T**NE		(26)
Rum1 :	T58	PP**T**PAK**T**PK		(27)

Every CPD contains a central phospho-threonine (except of Far1 and Clb6) followed by proline and a negative charge in the +4 position (glutamate or phosphosite). Large T is assigned to the mammalian CPDs, because it targets Fbw7. The CPDs for Notch and Presenilin have not been published yet and are putative. (*) indicates the amino acid position in full-length proteins before cleavage. Exceptions to the +4 negative charge rule are most CPDs of Sic1, Tec1, Hap1, CDC18 and Cig2 all of which contain multiple low-affinity degrons (not shown).

Because the fission yeast Fbw7 homologs, pop1 and pop2, have previously been shown to dimerize [28], we tested whether Fbw7 can also form dimers and how dimerization affects its functions. We identified a dimerization domain in the region common to all Fbw7 isoforms just upstream of the F-box. Fbw7 mutants that cannot dimerize are fully active toward cyclin E and c-Myc in turnover assays in cells, indicating that dimerization is not strictly required for Fbw7 function. However, we found that stable interactions between dimerization-deficient Fbw7 and cyclin E are largely impaired and that cyclin E turnover by monomeric Fbw7 completely depends on phosphorylated S384 – even by the Fbw7α isoform. Our results link Fbw7 dimerization to the negative charge in a CPD's +4 position and point to an additional level of complexity in substrate turnover by Fbw7.

## Results

### Identification of an Fbw7 dimerization domain

**Figure 1:**
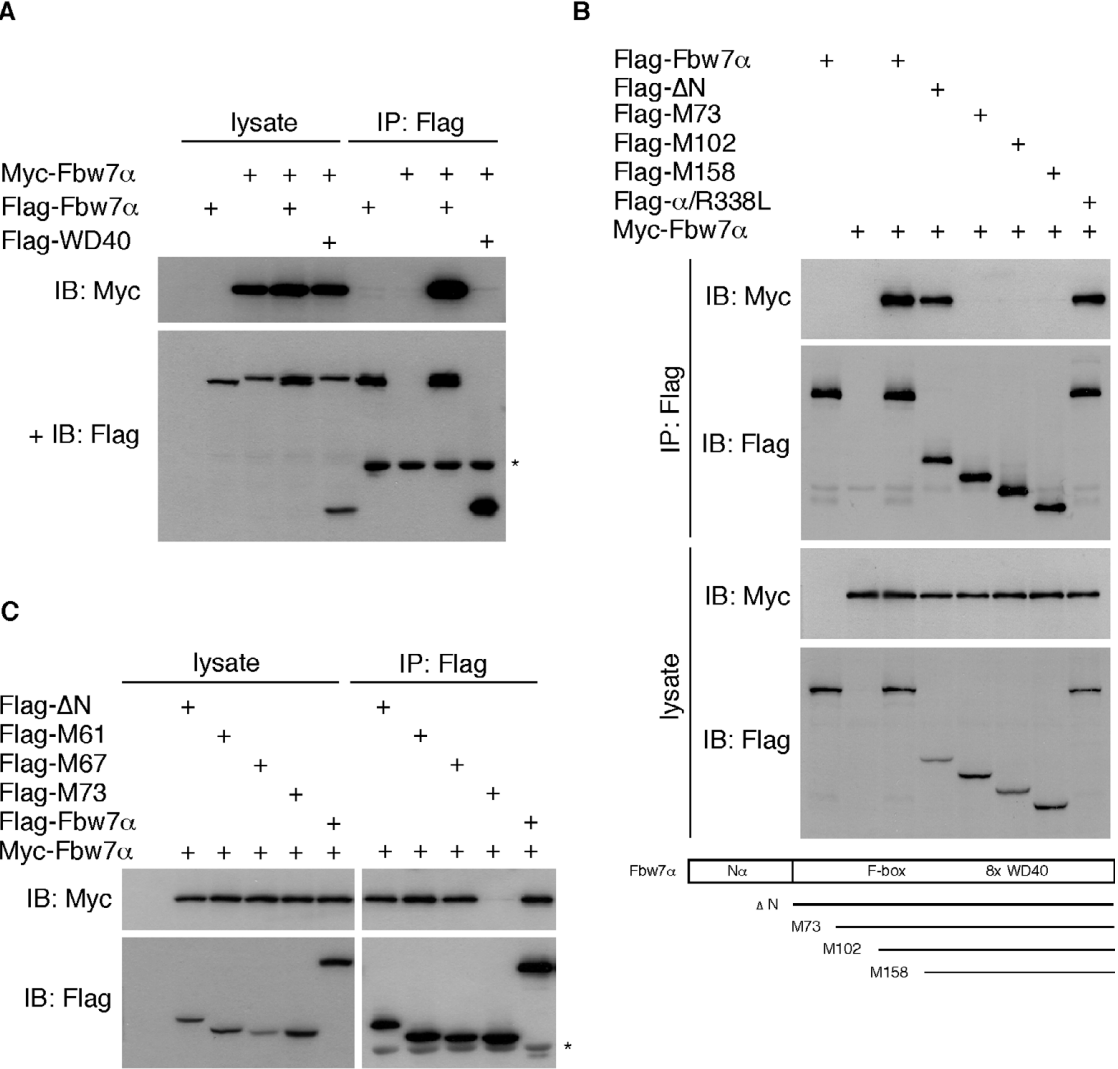
**Mapping of the Fbw7 dimerization domain (1)**. **A**: Fbw7α can form dimers. Flag- and Myc-tagged versions of Fbw7α as well as the Flag-tagged WD40 domain were transfected in 293A cells as indicated and analyzed for their interaction by immunoprecipitation with Flag antibody. Co-precipitated Myc-tagged Fbw7α was detected with 9E10 antibody (upper panel). The membrane was subsequently re-probed with Flag antibody (lower panel). Flag-Fbw7α corresponds to the lower band, Myc-Fbw7α to the upper band. The asterisk indicates the heavy chain of the Flag antibody used for immunoprecipitation. **B**: Analysis of N-terminal truncation mutants. Myc-tagged Fbw7α was co-expressed with Flag-tagged Fbw7α or various N-terminal truncation mutants as well as a point mutant of Fbw7α that no longer interacts with CPDs. Lysates were immunoprecipitated with Flag antibody and probed as indicated. ΔN refers to an N-terminal-less Fbw7 construct corresponding to the common region of Fbw7. All amino acid numbers reflect positions within the common region. The truncation mutants are depicted at the bottom. **C**: Fine-tuning of the dimerization domain beginning. The assay is similar as in A. The asterisk indicates the heavy chain of the Flag antibody used for immunoprecipitation.

In order to determine whether Fbw7 can form dimers, we generated Flag-tagged or Myc-tagged versions of Fbw7α. Both versions were transiently expressed in 293A cells and immunoprecipitates from cell lysates were analyzed for their interaction. The results demonstrated that Myc-tagged Fbw7α efficiently co-precipitated with Flag-tagged Fbw7α (Fig. 1A), while a truncated version of Fbw7 that only consists of the WD40 repeats did not interact with full length Fbw7α. Identical results were achieved in a reciprocal immunoprecipitation (anti Myc tag) from the same lysates (not shown). Therefore, like pop1 and pop2, Fbw7α can dimerize. In fact, dimerization may be a more common feature of F-box proteins, since β-TrCP has also been shown to form dimers [29].

We then attempted to define the region on Fbw7α necessary for dimerization. A panel of N-terminal truncation mutants was constructed and tested for interaction with full-length Fbw7α as above. This analysis revealed that a region common to all three Fbw7 isoforms (but lacking the isoform-specific N-terminus) was required to bind to full-length Fbw7 (Fig. 1B). However, the M73 truncation mutant (starting at methionine 73 of the common region) was incapable of binding to Fbw7α, suggesting that residues upstream of M73 are essential for dimerization. Substrate interaction is not required for Fbw7 dimerization, since a cancer-associated Fbw7 point mutant that no longer recognizes CPDs, still bound to wild-type Fbw7α (Fig. 1B, last lane).

**Figure 2:**
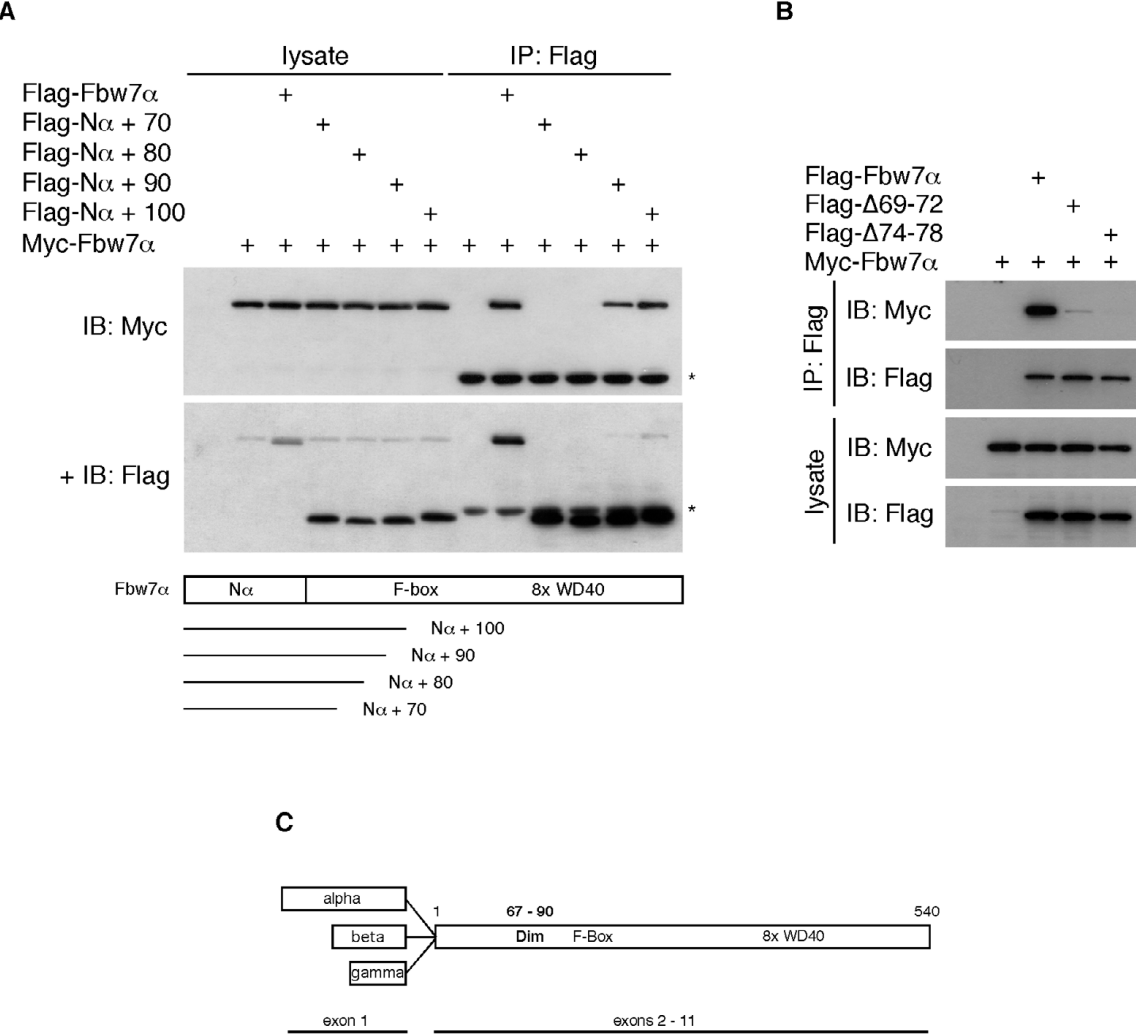
**Mapping of the Fbw7 dimerization domain (2)**. **A**: Analysis of C-terminal truncation mutants. Fbw7α was terminated after 70, 80, 90, and 100 amino acids of the common region (schematic below) and assayed as in A. The 9E10 blot (for detection of Myc-tagged Fbw7α) was subsequently probed with Flag antibody. **B**: Short deletion mutants eliminate dimerization. Same assay, except that two short deletion mutants were tested for their binding to full length Myc-tagged Fbw7α. The amino acid numbering of the deletions in full-length Flag-Fbw7α again corresponds to their positions in the common region. **C**: Schematic of Fbw7 splice forms including their WD40 repeats, F-box, and dimerization domain.

In order to more precisely define the dimerization domain, we generated several additional truncation mutants beginning just upstream of M73 and tested them in co-immunoprecipitation assays as above. This demonstrated that the M67 mutant (beginning at amino acid 67 of the common region) was still capable of binding to Fbw7α, suggesting that dimerization occurs via a domain immediately downstream of amino acid 67 (Fig. 1C). We next generated a panel of C-terminal truncation mutants to determine the extent of the dimerization domain. As expected from the data shown above truncating Fbw7α at amino acid 70 of the common region eliminated dimerization with full-length Fbw7α (Fig. 2A). Termination at amino acid 80 also failed to enable dimerization. However, terminating Fbw7α at amino acid 90 restored stable binding to full-length Fbw7α by about 50%, and was completely restored at amino acid 100 of the common region. Based on the analyses of the truncation mutants we then generated two adjacent deletion mutants in full-length Fbw7α just downstream of amino acid 67 (Δ69–72 and Δ74–78) and tested them for dimerization defects. Figure 2B clearly demonstrates that both mutants are severely impaired with regards to dimerization. The dimerization of an additional deletion mutant lacking amino acids 82 to 87 was also largely compromised (not shown). We conclude that Fbw7 dimerizes via an extended domain between amino acids 67 and at least 90 of the common region (see model, Fig. 2C).

**Figure 3:**
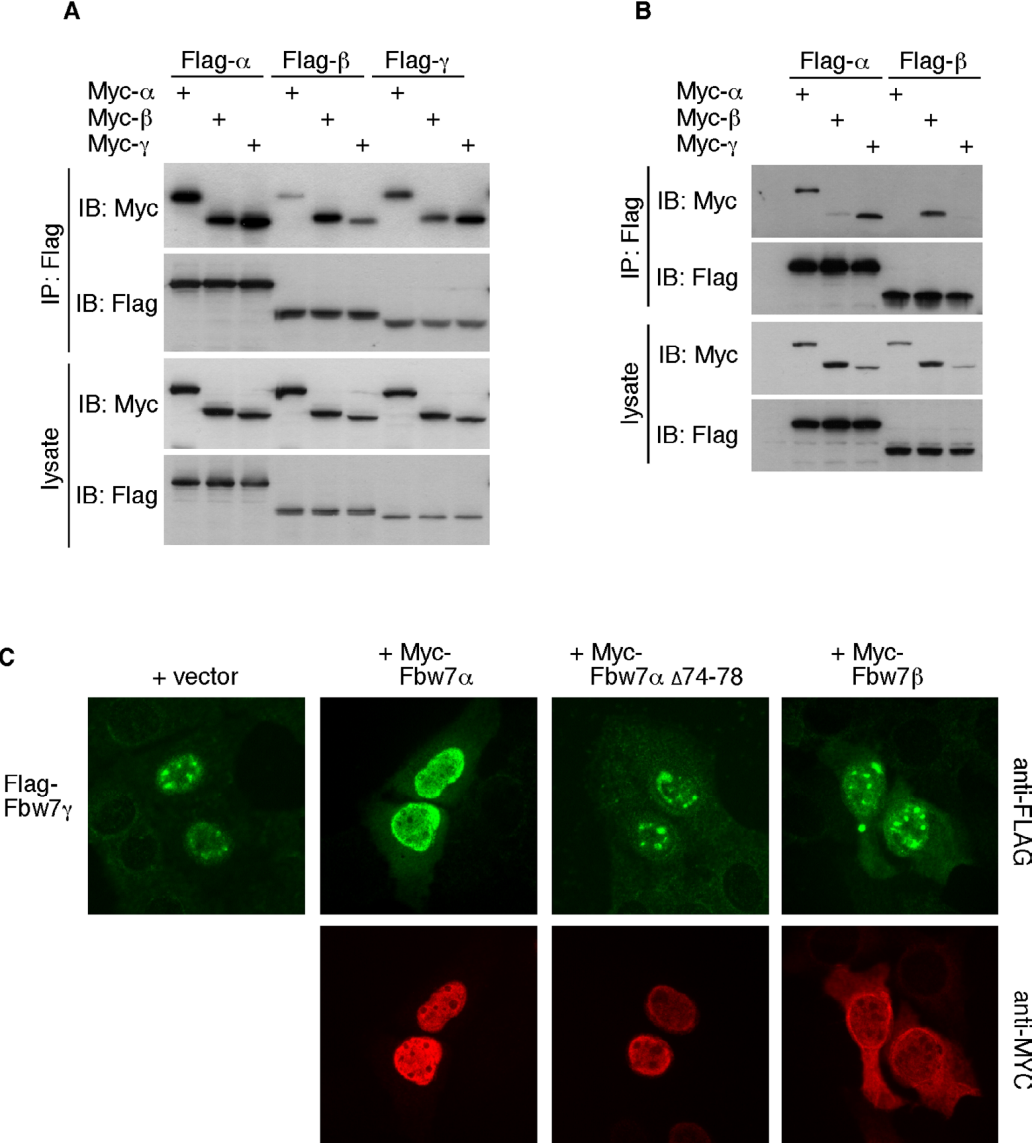
**Fbw7 isoforms interact in cells (1)**. **A**: Homo- and heterodimerization of Fbw7. Differentially tagged Fbw7 isoforms were transfected as indicated and lysates subjected to immunoprecipitations with Flag antibody. **B**: Fbw7α and Fbw7γ heterodimerize. Identical assay as in A, except 5-fold lower amounts of each isoform were expressed. **C**: Heterodimerization of Fbw7α and Fbw7γ occurs in cells. U2OS cells grown on cover slips were co-transfected with Flag-Fbw7γ and Myc-tagged versions of either Fbw7α, the Fbw7α dimerization mutant, or Fbw7β. Cover slips were fixed and co-stained with Flag and Myc antibodies.

Because we mapped the dimerization domain to the common region of Fbw7, we asked whether Fbw7β and Fbw7γ also homodimerize, and whether the different isoforms heterodimerize. Myc- and Flag-tagged versions of all three Fbw7 isoforms were analyzed for their interactions by co-immunoprecipitation. As expected both the Fbw7β and Fbw7γ isoforms could homodimerize (Fig. 3A). Interestingly, there was a clear preference for homo- over heterodimerization, most likely reflecting the differing sub-cellular localization of each isoform. Furthermore, some isoforms were able to form heterodimers, at least when overexpressed in 293A cells. Since vast overexpression can lead to leaking of the Fbw7 isoforms into different subcellular compartments, we repeated this experiment using 5-fold lower amounts of plasmid DNA. This demonstrated a more pronounced tendency for homo- over heterodimerization, in particular for cytoplasmic Fbw7β (Fig. 3B). However, nuclear Fbw7α and nucleolar Fbw7γ were still able to interact efficiently under these conditions.

### Fbw7 dimerization in cells

In order to determine whether heterodimerization may be a post-lysis artifact or actually occurs in cells, we analyzed these interactions by immunofluorescence of fixed cells. U2OS cells were grown on coverslips and transfected with each individual Flag-tagged Fbw7 isoform either alone or together with Myc-tagged Fbw7 isoforms in every heterodimeric combination. The localization of Flag-tagged Fbw7 was then assayed by immunofluorescence. As shown in figure 3C, co-expression of the nucleoplasmic Fbw7α protein caused the normally nucleolar Fbw7γ protein to become mislocalized to the nucleoplasm suggesting that they interact in cells, and this was dependent on an intact dimerization domain of Fbw7α. In contrast, co-expression of cytoplasmic Fbw7β did not relocalize Fbw7γ. Consistent with the preference for homodimerization observed in figures 3A and 3B, Fbw7α and Fbw7β were largely unaffected by co-expressing any other isoform (not shown).

**Figure 4:**
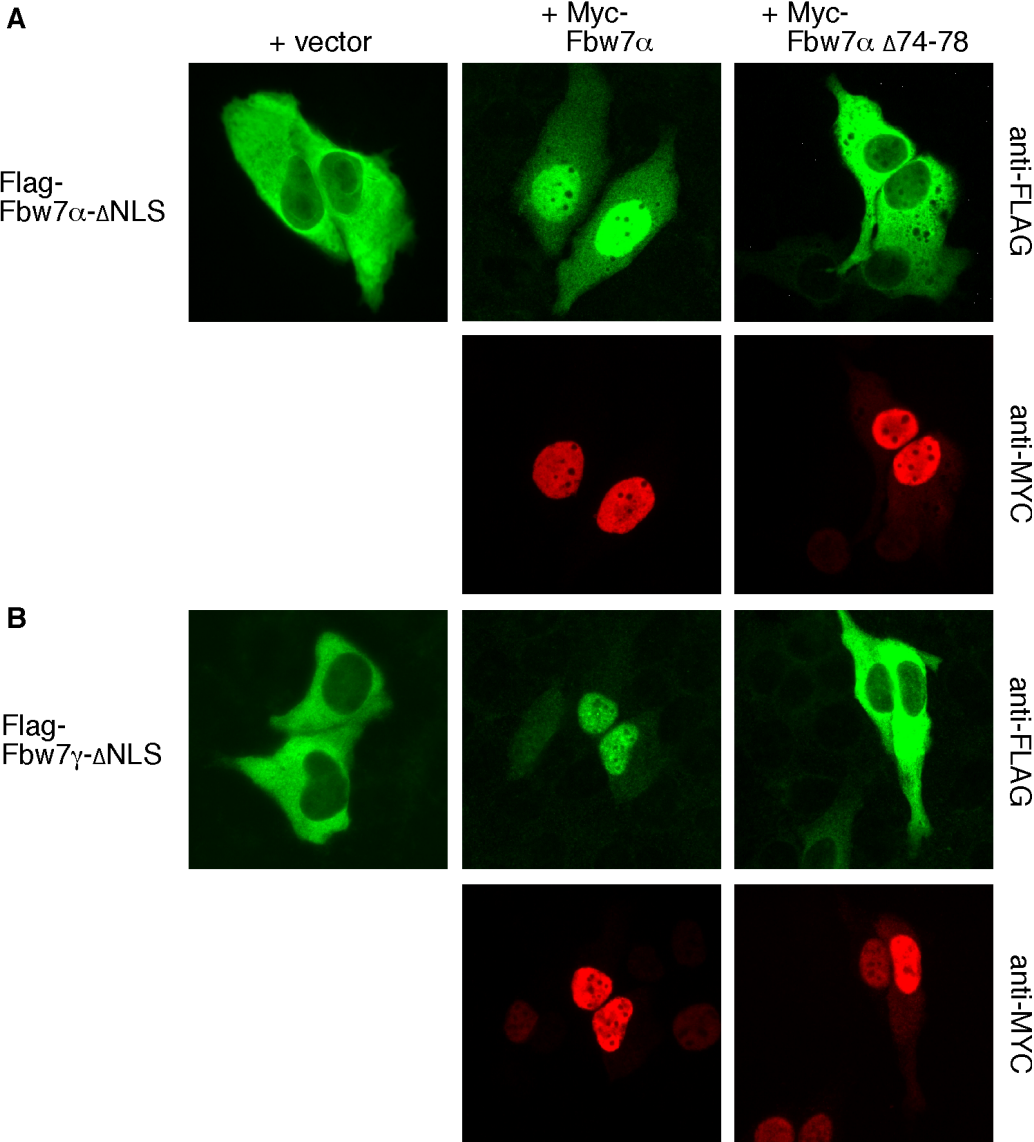
**Fbw7 isoforms interact in cells (2)**. Cytoplasmic versions of Fbw7α (**A**) and Fbw7γ (**B**) lacking their according nuclear localization signals (NLS) were co-expressed with Myc-tagged Fbw7α or the dimerization mutant and cover slips co-stained with Flag and Myc antibodies. All images in are representatives of at least three independent experiments and were also carried out in 293A cells (not shown).

We also attempted to examine homodimerization with this assay. To this end, we made use of our previously described localization mutants that place both Fbw7α and Fbw7γ into the cytoplasm due to deletion of their nuclear localization signals (NLS)[13]. Remarkably, co-expression of Fbw7α relocalized both NLS mutants into the nucleoplasm in a dimerization-dependent fashion, clearly demonstrating their ability to interact with one another *in situ *(Fig. 4). These experiments were also performed in 293A cells with similar results (not shown). Taken together, all three Fbw7 isoforms can homo- and partially heterodimerize in intact cells, at least when expressed ectopically. This observation may have implications for the general compartmentalization of the endogenous Fbw7 isoforms (see discussion).

### Dimerization is not strictly required for Fbw7 function

**Figure 5:**
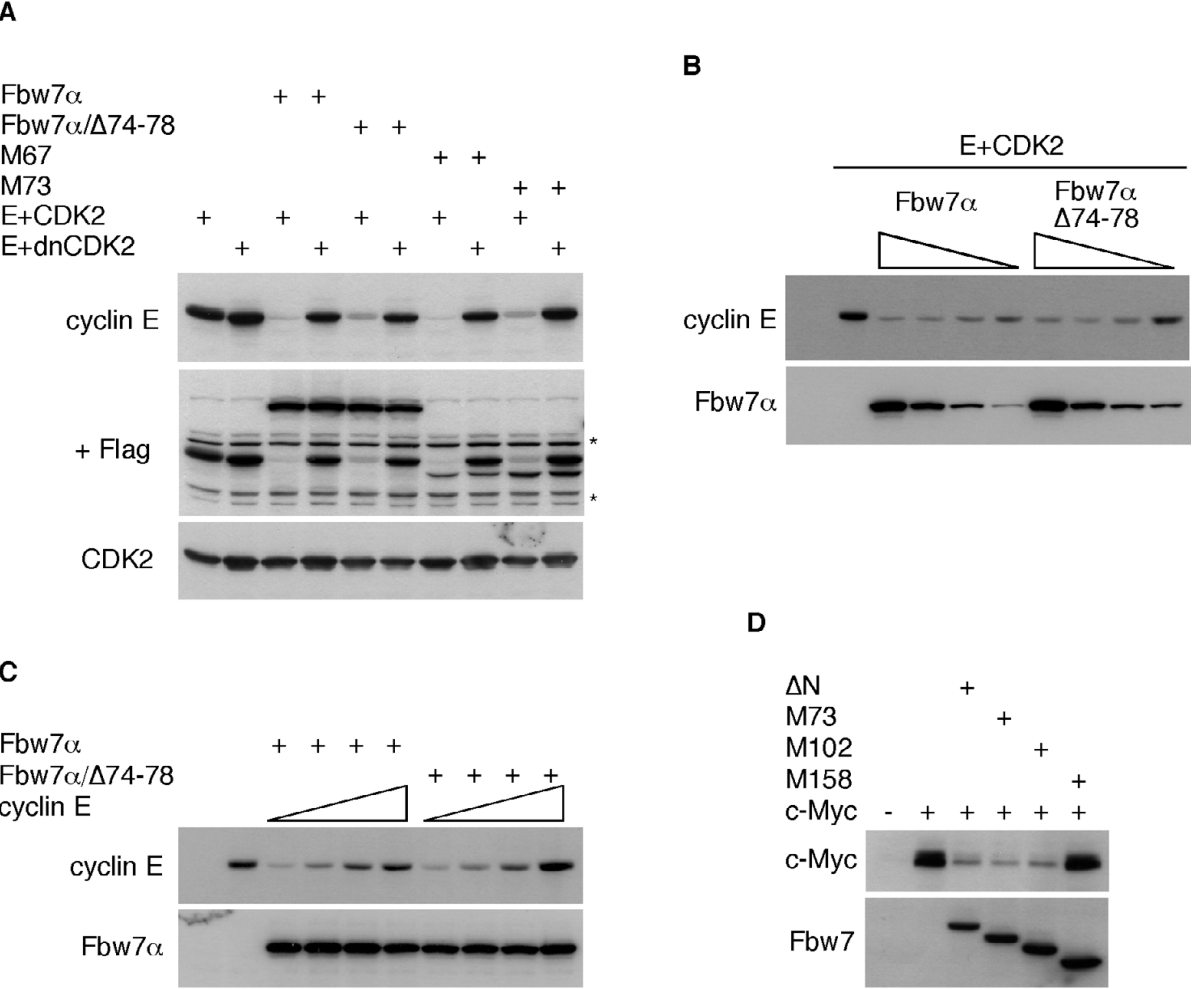
**Monomeric Fbw7 is active**. **A**: Turnover of cyclin E by dimerization mutants of Fbw7. 293A cells were transfected either with active cyclin E (wild-type CDK2) or inactive cyclin E (dominant-negative CDK2) together with various versions of Fbw7 as indicated. Steady-state protein abundance of cyclin E was monitored by immunoblotting (upper panel). The same membrane was subsequently blotted for Fbw7 and CDK2 (middle panel). The asterisks mark cross-reactions from the 12CA-5 antibody used to detect HA-tagged CDK2. A shorter exposure of CDK2 was chosen and is shown in the lower panel. **B**: Cyclin E turnover at altered Fbw7 stoichiometry. 0.5 μg of cyclin E plasmid was co-expressed with titrated amounts of either wild-type or dimerization-deficient Fbw7α (3, 1, 0.3, and 0.1 μg, respectively) and blotted for steady-state protein abundance as indicated. **C**: Cyclin E turnover by Fbw7 at altered stoichiometry. 2 μg of Fbw7 plasmid or the non-dimerizing mutant were co-expressed with increasing amounts of cyclin E (0.5, 1, 2, and 4 μg) and protein levels analyzed as above. In both experiments 2 μg of CDK2 were co-expressed (not shown). **D**: Fbw7 dimerization is not required for c-Myc degradation. c-Myc and Fbw7 truncation mutants were co-transfected as indicated and their protein levels analyzed by western blotting. ΔN refers to N-terminal-less Fbw7 and corresponds to the common region of Fbw7. All truncation mutants start at the indicated methionines within the common region. The M158 mutant no longer contains the F-box and is therefore inactive.

We next tested whether Fbw7 dimerization mutants are capable of promoting cyclin E and c-Myc turnover. Cyclin E and CDK2 were co-expressed with either wild-type Fbw7α or dimerization mutants in 293A cells and steady-state levels of cyclin E were analyzed by western blotting. As shown in figure 5A, both the non-dimerizing deletion mutant (Δ74–78) and the M73 truncation mutant were able to degrade cyclin E similar to wild-type Fbw7. We did not observe any significant impairment of the dimerization mutant compared to wild-type Fbw7α, even upon titration of either cyclin E or Fbw7 (Fig. 5B and 5C). Dimerization-defective Fbw7 also promoted c-Myc turnover (Fig. 5D) and bound well to SV40 Large T (not shown and [21]). Therefore, at least in these types of assays, Fbw7 dimerization is not strictly required for substrate degradation.

### Conditional requirement for Fbw7 dimers

**Figure 6:**
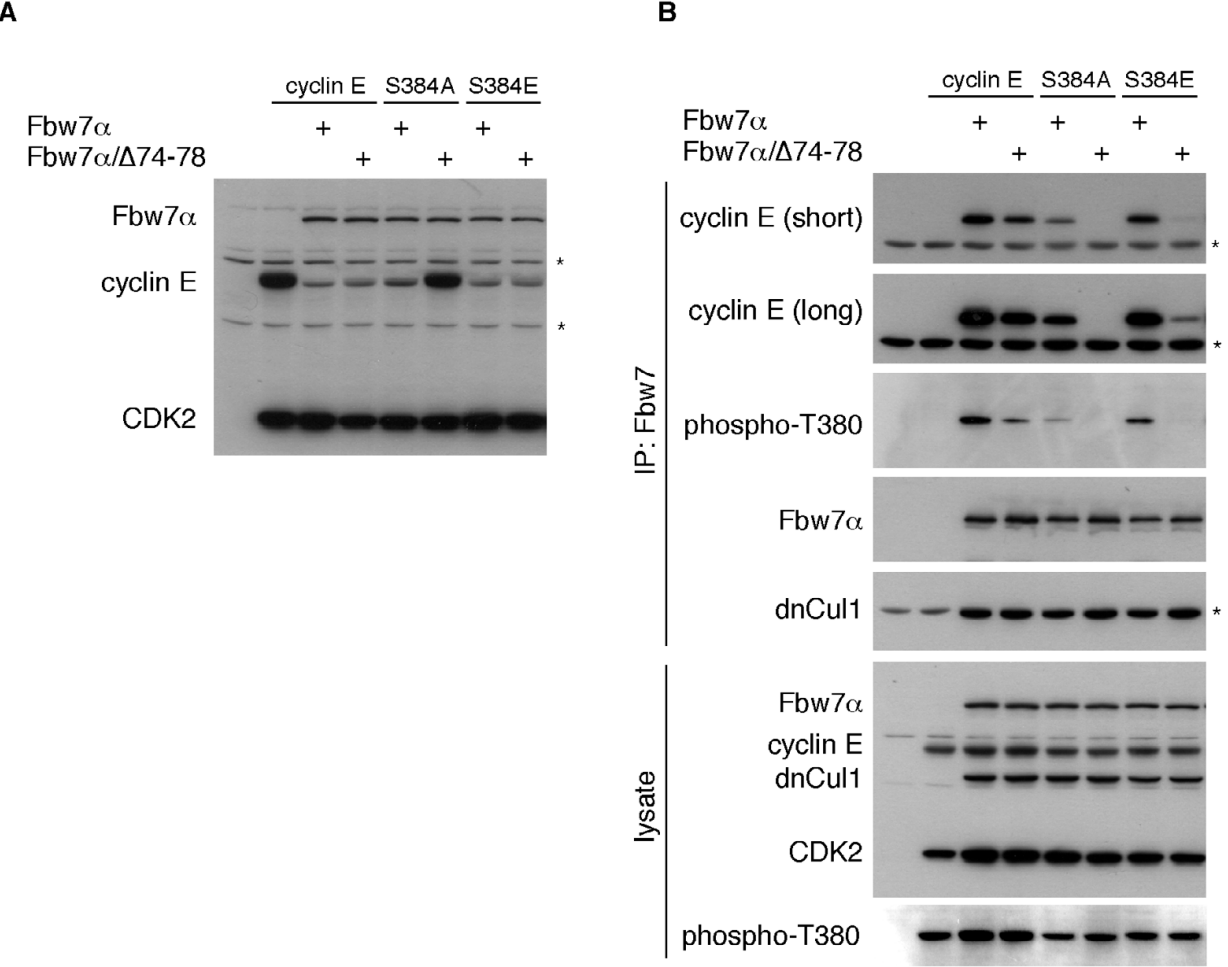
**A role for Fbw7 dimerization**. **A**: Dimerization-deficient Fbw7 strictly requires S384 phosphorylation for cyclin E turnover. Cyclin E/CDK2 or two S384 mutants of cyclin E (S384A and S384E) were co-transfected into 293A cells together with either wild-type or non-dimerizing Fbw7α as indicated. Lysates were probed for the steady-state expression of these proteins with an antibody cocktail. The asterisks indicate cross-reactions. **B**: Analysis of stable interaction between cyclin E and Fbw7α. 293A cells were transfected as indicated together with dominant-negative Cul1. Binding of Fbw7α to cyclin E was assayed by co-immunoprecipitation anti Flag. dnCul1 co-migrates with the Flag antibody heavy chain. The asterisks indicate heavy chain cross-reactions. The upper panel lysate membrane was incubated with a cocktail of antibodies to detect all relevant proteins.

The requirement for S384 phosphorylation in cyclin E turnover by Fbw7α is conditional and depends upon Fbw7 stoichiometry [20]. We therefore tested whether S384 manipulation would affect cyclin E's binding and turnover by Fbw7 dimerization mutants. Wild-type cyclin E or S384 mutants changed to alanine or glutamate were expressed in 293A cells together with either Fbw7α or the dimerization mutant. Steady-state abundance of cyclin E was then analyzed by western blotting. Figure 6A demonstrates that wild-type cyclin E and the S384E mutant are both efficiently degraded by Fbw7α and by the dimerization mutant. As expected, Fbw7α was also able to eliminate the S384A mutant at this stoichiometry. However, the S384A mutant was completely resistant to non-dimerizing Fbw7α (Fig. 6A), and this was independent of the stoichiometry between the S384A and Fbw7 mutants (not shown).

In addition to its potential priming role for GSK3, S384 phosphorylation participates in the formation of the T380 CPD and is likely to mediate direct contacts with Fbw7 [19]. We therefore directly tested the role of Fbw7 dimerization in binding to cyclin E and the S384 mutants by co-immunoprecipitation. In order to capture this transient interaction we co-expressed a dominant-negative version of Cul1, an essential core component of SCF complexes, in an otherwise identical experiment as in figure 6A. This prevents cyclin E ubiquitination and degradation and allows stable binding of cyclin E to Fbw7 [20]. Lysates were immunoprecipitated against Fbw7α or the dimerization mutant and western blotted for co-immunoprecipitated cyclin E (Fig. 6B). The results confirmed that the stable interaction between the S384A mutant and Fbw7α is largely impaired compared with wild-type cyclin E. This is not simply due to decreased T380 phosphorylation of this mutant (see lysate). In fact, the extent of T380 phosphorylation is comparable between the S384A and S384E mutants, yet only S384E is efficiently bound to Fbw7. This supports the notion that a negative charge in +4 directly contributes to Fbw7 binding.

**Figure 7:**
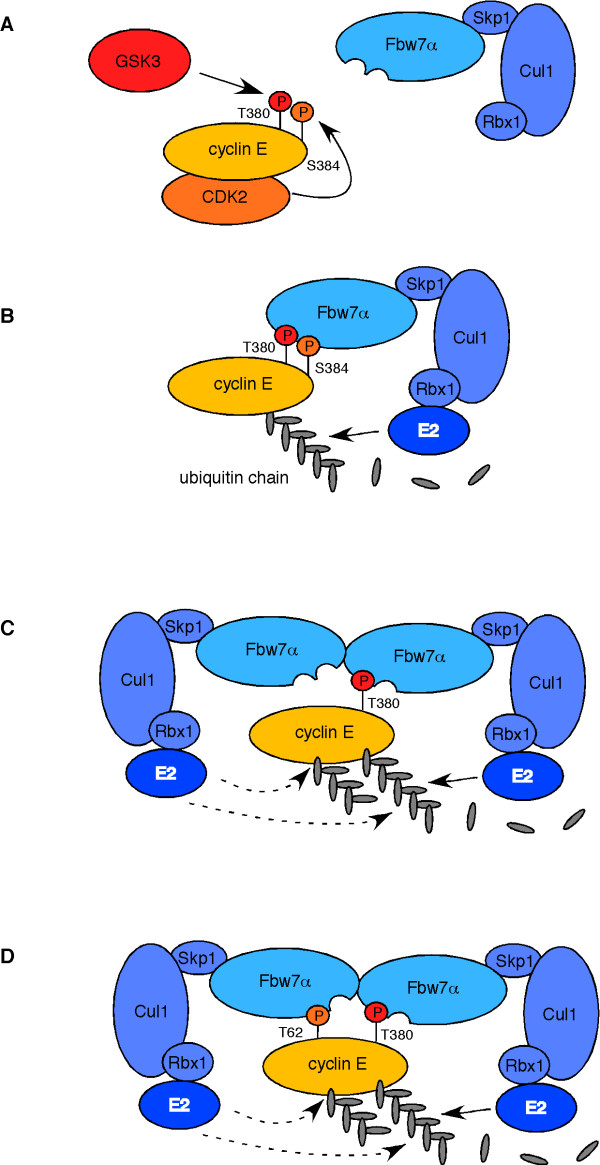
**Model for role of Fbw7α dimerization**. **A**: Cdk2 (S384) and GSK3 (T380) prime cyclin E for destruction. The hyper-phosphorylated T380/S384 degron has high affinity for monomeric Fbw7α, which engages the remainder of the SCF to initiate cyclin E's ubiquitination by an E2 enzyme (**B**). **C**: Hypo-phosphorylated cyclin E that lacks S384 phosphorylation within the T380 phospho-degron can still be degraded by Fbw7α. However, this destruction pathway requires Fbw7 dimerization. Dimerization recruits a second E2 ubiquitin conjugating enzyme (E2) that may help elongating the same ubiquitin chain or may initiate another chain. Alternatively, the second Fbw7α component of the dimer may actually contact another degron within cyclin E, possibly the T62 degron, and may help stabilizing the substrate interaction.

Dimerization-deficient Fbw7α, on the other hand, was partly compromised in binding to wild-type cyclin E and completely lost its ability to interact with the S384A mutant (Fig. 6B), even on very long exposures (not shown). Surprisingly, S384E failed to restore efficient binding to the Fbw7α dimerization mutant despite the finding that the S384E mutant is degraded by monomeric Fbw7 (compare Figs. 6A and 6B). The reason for this discrepancy is unclear, but probably reflects differences in contact formations of phospho-serine versus glutamate with the WD40 repeats of Fbw7. The implications of our findings are summarized in figure 7.

## Discussion

We have shown that human Fbw7 can form dimers and mapped the dimerization domain to a region just upstream of the F-box. Since this domain is preserved in all Fbw7 splice variants, Fbw7 may, in principle, be able to form both homo- and heterodimers. However, we and others have demonstrated previously that the Fbw7 isoforms localize to distinct subcellular compartments [12,13,20]. Due to the absence of suitable antibodies, all Fbw7 localization studies relied on overexpressed tagged Fbw7, and all conclusions were drawn from the expression of a single Fbw7 isoform at a time. Here we show that, at least upon co-overexpression, some isoforms can interact in cells and alter their localization through heterodimerization. This suggests that, in addition to the cis-acting signals that normally direct them to their locations, the stoichiometry of the endogenous Fbw7 isoforms and the extent of heterodimerization may influence their compartmentalization. It is unclear to what extent (hetero)dimerization among the endogenous Fbw7 isoforms occurs. Due to their low abundance, we are currently unable to detect the endogenous Fbw7 proteins and are restricted to studying ectopically expressed Fbw7. One intriguing consequence of heterodimerization is that one Fbw7 isoform may be able to regulate the activity of another isoform by directing it to another cellular address. For instance, the extent of heterodimerization between Fbw7α and Fbw7γ may regulate the amount of nucleolar Fbw7γ and, hence, the activities of nucleolar substrates.

Besides potentially affecting isoform localization, dimerization may directly regulate other functional aspects of Fbw7. For instance, since the dimerization domain is adjacent to the F-box, we initially tested the idea that dimerization may prevent the interaction with Skp1 and, hence, inactivate Fbw7. However, we found no evidence for this, since Skp1 bound indistinguishably to monomeric and dimerized Fbw7 (not shown). Instead, we uncovered a requirement of Fbw7 dimerization for the regulation of certain substrates. Our results establish a link between Fbw7 dimerization and the presence of a negative charge in the +4 position of cyclin E's T380-CPD. The implications of our findings suggest differential modes of action by Fbw7 and sub-divide Fbw7 targets into three groups. First are substrates that always provide the negative charge in +4 via glutamates, aspartates, or constitutive (priming) phosphorylation. This subgroup may not rely on Fbw7 dimerization for their turnover. Second are substrates that sometimes, perhaps conditionally, provide this charge and may switch their CPD affinity in response to environmental cues. Cyclin E is an example of this group and can be degraded independent of Fbw7 dimerization when S384 is phosphorylated, but becomes dependent on S384 phosphorylation for monomeric Fbw7 (see model in figure 7). Also c-Myc may belong to this subgroup, since it has been suggested that S62 became de-phosphorylated after having served as primer for T58 phosphorylation [30]. However, in our assays c-Myc regulation by Fbw7 is not dependent on Fbw7 dimers (Fig. 5D). Finally, some substrates may never provide this charge and therefore are completely dependent on Fbw7 dimerization for their degradation. Such CPDs probably mediate weaker interactions with Fbw7 and may entirely rely on cooperative effects together with other (weak) CPDs. The activities of these substrates may in part be controlled by the state of Fbw7 dimerization and are possibly subjected to an additional level of regulation, if dimerization itself was controlled.

Sic1 requires at least six phosphorylated CPDs for optimal destruction by CDC4, each of which are flawed by the absence of the +4 negative charge, the presence of non-favorable basic amino acids, or both [11,16]. Therefore, the low-affinity CPDs of Sic1, like cyclin E that is not phosphorylated on S384, may entirely depend on CDC4 dimerization for efficient turnover. Indeed, recent studies have shown that Sic1 degradation requires CDC4 dimerization (Mike Tyers personal communication). Why low affinity CPDs require F-box protein dimerization remains unclear, but perhaps their affinity is so low that the recruitment of several SCF complexes is required for efficient substrate degradation. Dimerized Fbw7 (or CDC4) may simply be twice as efficient in substrate ubiquitination, because two SCF complexes can recruit two E2 ubiquitin ligases, and this may be necessary for the turnover of low-affinity substrates.

Several mechanistic ideas arise from our observations. One possibility is that dimerized Fbw7 binds to two different CPDs concurrently to increase substrate affinity and facilitate more efficient ubiquitination of its substrate. This model would be a plausible explanation to account for the more transient interactions of Fbw7/CDC4 with low-affinity CPDs and is depicted in figure 7D. Alternatively, a dimer may bind to a single CPD via one of the dimer moieties while the other one simply recruits a second E2 enzyme, perhaps for more efficient ubiquitination (Figure 7C). Moreover, a dimerized SCF might elongate the same ubiquitin chain, act on two different chains, or perhaps several dimers elongate one single ubiquitin chain when bound to different CPDs. All of these models may apply in a substrate-specific fashion or even for different CPDs within the same substrate.

## Conclusion

Our results suggest that Fbw7's affinity for certain substrates underlies yet another level of regulation that links the negatively charged +4 position of CPDs and Fbw7's ability to form dimers. We propose that weak CPDs that do not contain the + 4 charge must either cooperate with another (weak?) CPD or bring a second SCF to the same low-affinity CPD for efficient substrate destruction via Fbw7 dimerization.

## Methods

### Cell culture, transfections, plasmids

U2OS (human osteosarcoma) and HEK-293A (human embryonic kidney) cell lines were cultured under standard conditions in DMEM containing 10% FCS. For transient transfections experiments, cells were seeded into 6 cm dishes and transfected the next day by Ca-precipitation overnight at 30 to 40% confluence. 24 hrs after washing and replacing the media cells were harvested either for protein extracts or immunostaining. Typically, we express 1/10 of the plasmid amount of pFlag-Fbw7α (0.3 μg) compared to Fbw7β and Fbw7γ (3 μg each). The *in vivo *Fbw7-driven cyclin E turnover assay was performed as described [19]. All point mutants and deletions were introduced by the Quick Change method (Stratagene) and confirmed by sequencing. The deletions Δ69–72 and Δ74–78 correspond to amino acids EWLK and FQSWS, respectively. Truncation mutants were generated by PCR and sequenced. All amino acid numbers indicate their positions within the common region of Fbw7.

### Immunofluorescence, immunoprecipitation, immunoblotting

For immunofluorescence, cells were seeded and transfected on glass coverslips. Slips were fixed with ice-cold Methanol/Acetone (1:1) for 5 min, air-dried and immunostained with primary antibody. After washing with PBS, slips were incubated with secondary FITC-coupled anti-mouse antibody, washed, dried and mounted. For double stainings mouse Flag-tag and rabbit Myc-tag antibodies were mixed and visualized with FITC- and dopamine-coupled secondary antibodies. All protein extracts were made with Tween 20 lysis buffer [19]. Protein expression was determined by standard procedures (SDS-PAGE, western blot, immunoblot). Protein complexes were analyzed by immunoprecipitation. The following primary antibodies were used in this study: anti-Flag for Fbw7 (Sigma, M2), anti-HA for CDK2 and dnCul1 (12CA-5), anti-c-Myc (Santa Cruz, sc-N262), anti-Myc-tag for cyclin E and Fbw7 (9E-10 and Cell Signaling #2278), anti-pT380 for phospho-cyclin E (Santa Cruz, sc-12917-R).

## Competing interests

The author(s) declare that they have no competing interests.

## Authors' contributions

MW performed all experiments and wrote this manuscript. BEC edited the manuscript. Both authors have approved the final version of this manuscript.

## Note added in proof

While this manuscript was under revision a similar study was published by Zhang and Koepp (Mol Cancer Res December 2006).
